# Supplementary material for: Expression and Activity of TRPA1 and TRPV1 in the Intervertebral Disc: Association with Inflammation and Matrix Remodeling
Source: Int J Mol Sci. 2019 Apr 10;20(7):1767. doi: 10.3390/ijms20071767 (PMC6480240; doi:10.3390/ijms20071767)
Supplement: Supplementary file 1 [file ijms-20-01767-s001.pdf]

Supplementary figures

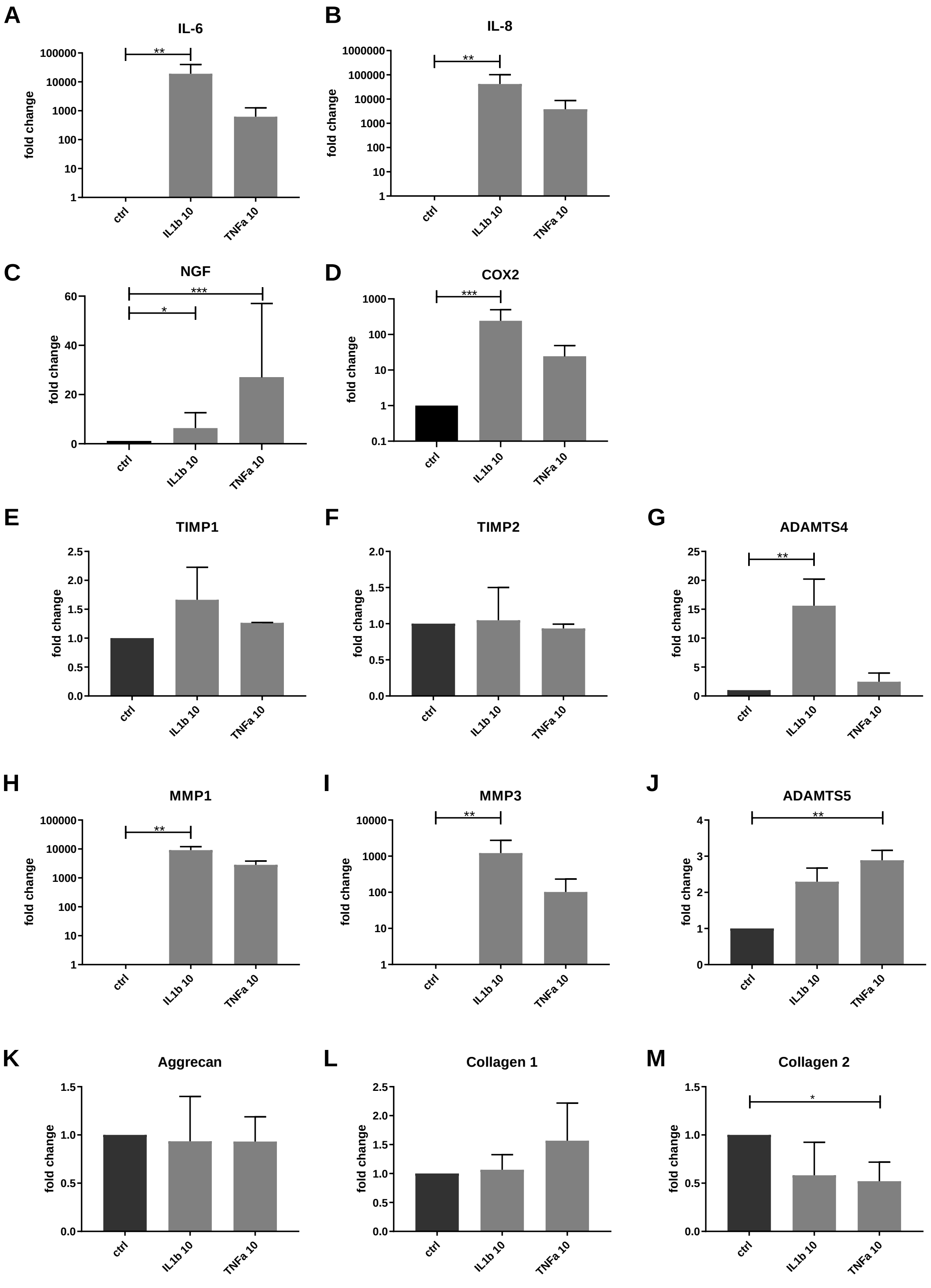

Supplementary figure S1: Gene expression of inflammation mediators, ECM remodeling enzymes and ECM genes in IVD cells treated with 5 and 10 ng/mL IL-1β or TNF-α. *Gene expression of inflammation mediators* ***(A)*** *IL-6,* ***(B)*** *IL-8, pain mediators* ***(C)*** *NGF,* ***(D)*** *COX-2 and ECM remodeling enzymes,* ***(E)*** *TIMP1 and* ***(F)*** *TIMP2,* ***(G)*** *ADAMTS4,* ***(H)*** *MMP1,* ***(I)*** *MMP3 and* ***(J)*** *ADAMTS5. Gene expression of extracellular matrix genes* ***(K)*** *Aggrecan,* ***(L)*** *Collagen I and* ***(M)*** *Collagen II. Graphs show*

2^{-ddCt} (mean ± SD, n = 5). Asterisks indicate statistical significance ($p < 0.05$, Kruskal-Wallis test). Fold change of IL-6, IL-8, COX-2, MMP-1 and MMP-3 is shown in log scale.

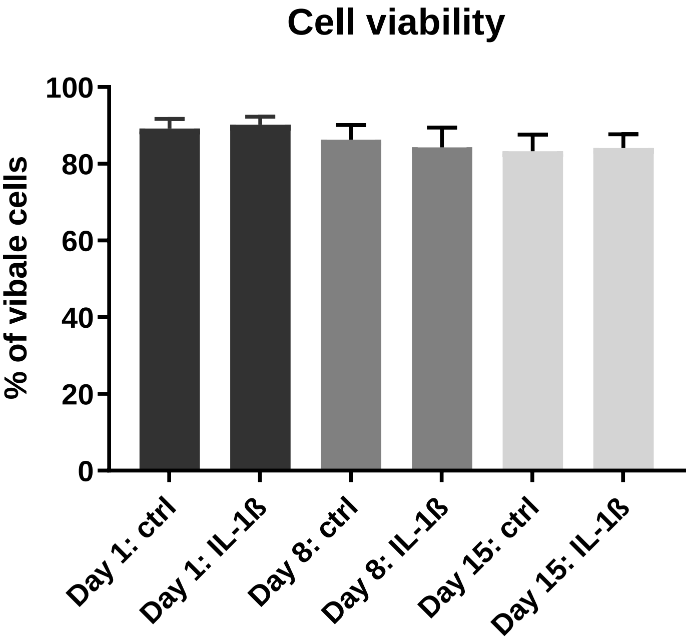

Supplementary figure S2. *Cell viability of IVD cells cultured in 3D alginate beads for 15 days. Graph shows % of viable cells (mean ± SD, n = 5). Asterisks indicate statistical significance ($p < 0.05$, Kruskal-Wallis test).*

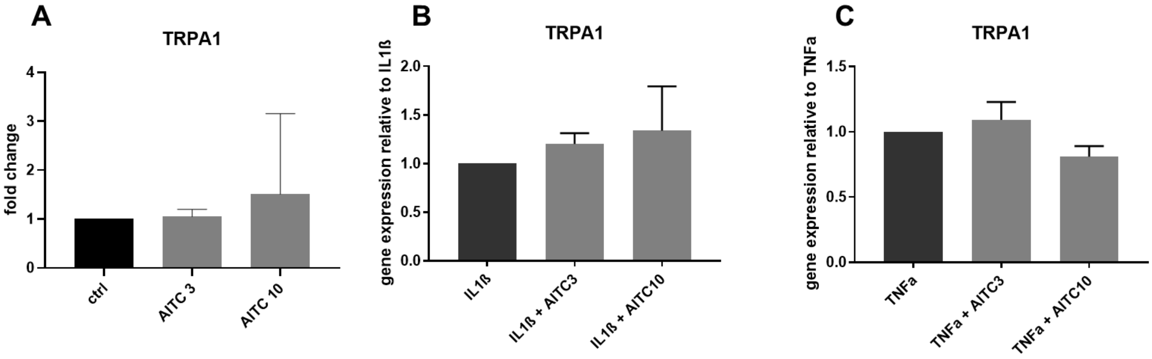

Supplementary figure S3. *Gene expression of TRPA1 in IVD cells treated with IL-1β and TNF-α with and **(A)** without with TRPA1 agonist allyl isothiocyanate (AITC, 3 and 10 µM) ± **(B)** 10 ng/mL IL-1β or **(C)** TNF-α. Graphs show gene expression as fold change and calculated relative to IL-1β or TNF-α treatment (mean ± SD, n = 3). Asterisks indicate statistical significance ($p < 0.05$, Kruskal-Wallis test)*
